# Supplementary material for: Wochenende — modular and flexible alignment-based shotgun metagenome analysis
Source: BMC Genomics. 2022 Nov 11;23:748. doi: 10.1186/s12864-022-08985-9 (PMC9650795; doi:10.1186/s12864-022-08985-9)
Supplement: Supplementary file 1 — Additional file 1: Supplementary Figure S1. Genome coverage plot of B.intestinalis reported by Wochenende. Wochenende did not misclassify B. subtilis in the mock community by Sui et al (SRSRR11207337) [29], but rather detected B. intestinalis with a high and evenly distributed coverage. Indeed all the other tools also reported B. intestinalis instead of B. subtilis. Supplementary Figure S2. Wochenende analysis of an alternative long-read Zymo Even DNA mock community. The mock community was sequenced on an Oxford Nanopore GridION sequencer by the laboratory of Nick Loman (https://github.com/LomanLab/mockcommunity). To our knowledge, the other tested tools are not able to analyze these long reads appropriately. The dataset and analysis is plausible yet suboptimal, as none of the species was found within their expected range, though Enterococcus faecalis, Staphylococcus aureus and Listeria monocytogenes come close. Lactobacillus fermentum is present at higher abundance than the expected range. B.subtilis is again underrepresented, similarly to the results from short read data presented in Figure 2 [29]. Supplementary Figure S3. A heat tree automatically produced by our tool Haybaler using the R package metacoder. The taxonomy of this fairly typical airway metagenome is illustrated succinctly and is useful for rapid initial comparative analyses across samples. Rothia mucilaginosa and Haemophilus influenzae dominate, though diverse Streptococcus and several Veillonella and Prevotella species are also present. Supplementary Figure S4. Reads from a skin swab were mapped to a fungus from the Wochenende reference genome. These reads were mapped with high mapping quality to all Candida tropicalis supercontigs, providing a rare example of a fungus in this metagenome. Fungi are generally difficult to reliably locate in metagenomes because of low abundance, poor reference genomes and wet lab sampling bias due to their highly resistant physical structures. Fungi remain rare in ou [file 12864_2022_8985_MOESM1_ESM.docx]

# Supplementary Material


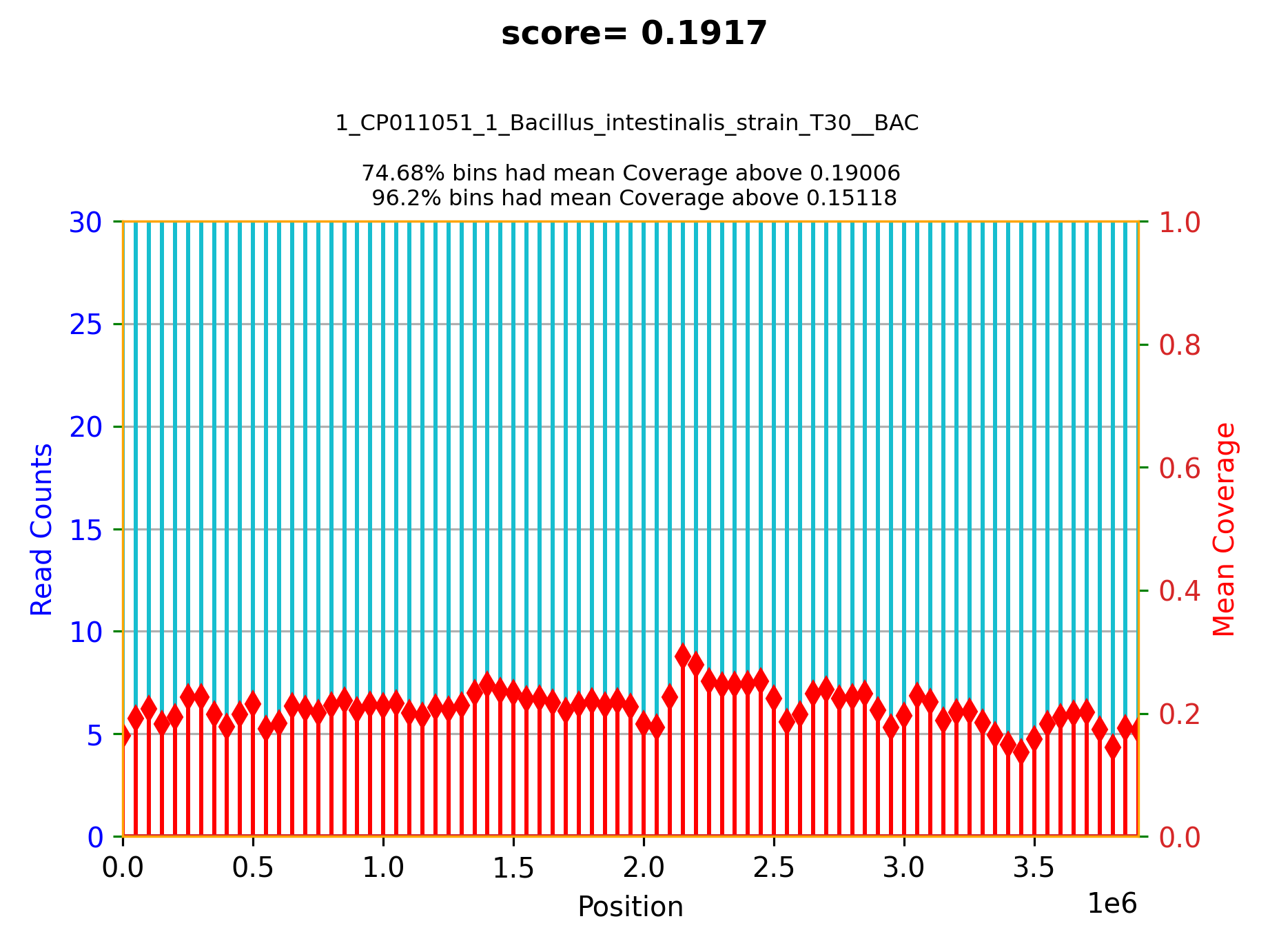


**Supplementary Figure S1 Genome coverage plot of *B. intestinalis* reported by Wochenende.** Wochenende did not misclassify *B. subtilis* in the mock community by Sui et al (SRSRR11207337) [[29]](https://paperpile.com/c/SFxJa8/u5yCF), but rather detected *B. intestinalis* with a high and evenly distributed coverage. Indeed all the other tools also reported *B. intestinalis* instead of *B. subtilis*.


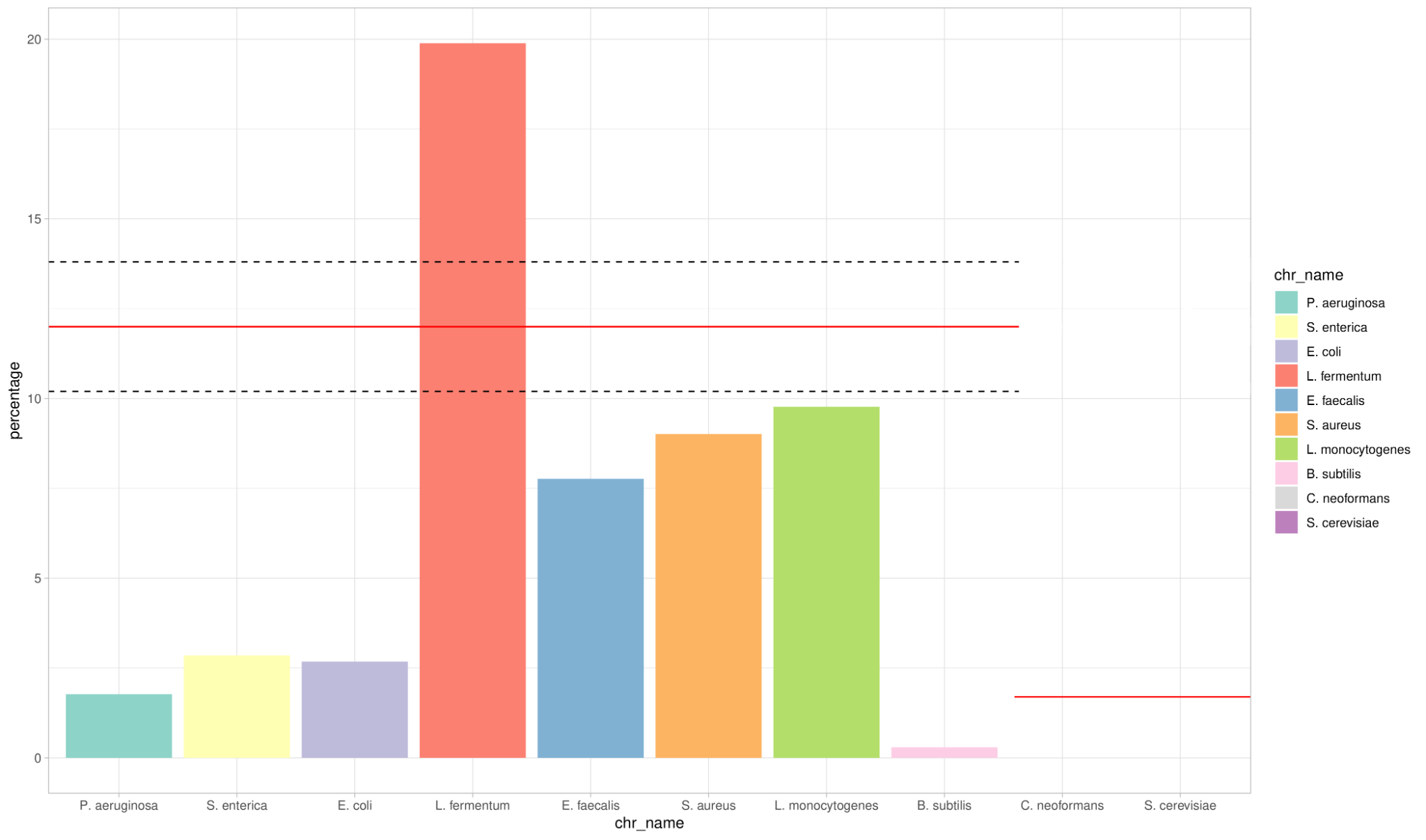


**Supplementary Figure S2** **Wochenende analysis of an alternative long-read Zymo Even DNA mock community.** The mock community was sequenced on an Oxford Nanopore GridION sequencer by the laboratory of Nick Loman (<https://github.com/LomanLab/mockcommunity>). To our knowledge, the other tested tools are not able to analyze these long reads appropriately. The dataset and analysis is plausible yet suboptimal, as none of the species was found within their expected range, though *Enterococcus faecalis*, *Staphylococcus aureus* and *Listeria monocytogenes* come close. *Lactobacillus fermentum* is present at higher abundance than the expected range. *B. subtilis* is again underrepresented, similarly to the results from short read data presented in Figure 2 [[29]](https://paperpile.com/c/SFxJa8/u5yCF).


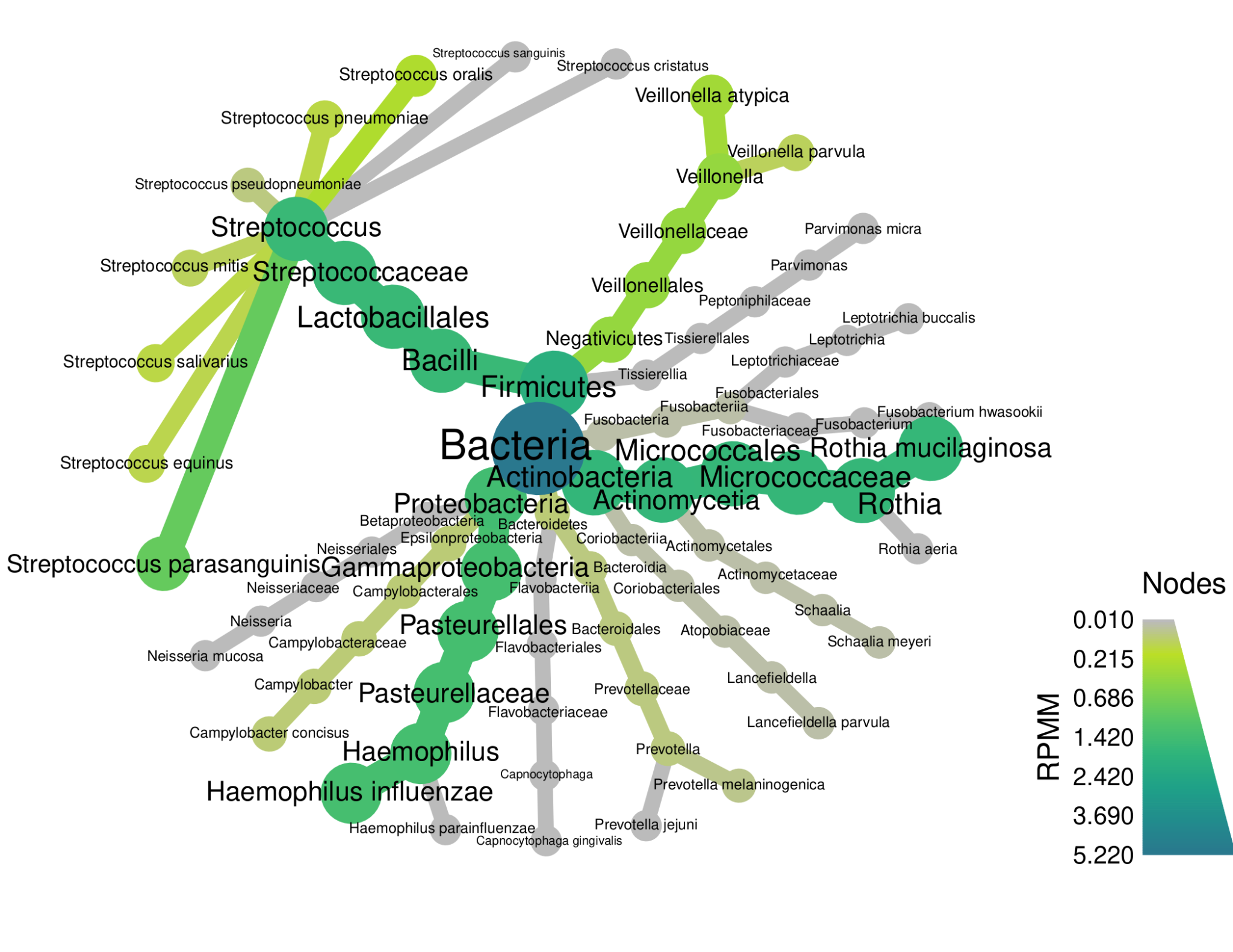


**Supplementary Figure S3** **A heat tree automatically produced by our tool Haybaler using the R package metacoder.** The taxonomy of this fairly typical airway metagenome is illustrated succinctly and is useful for rapid initial comparative analyses across samples. *Rothia mucilaginosa* and *Haemophilus influenzae* dominate, though diverse Streptococcus and several Veillonella and Prevotella species are also present.


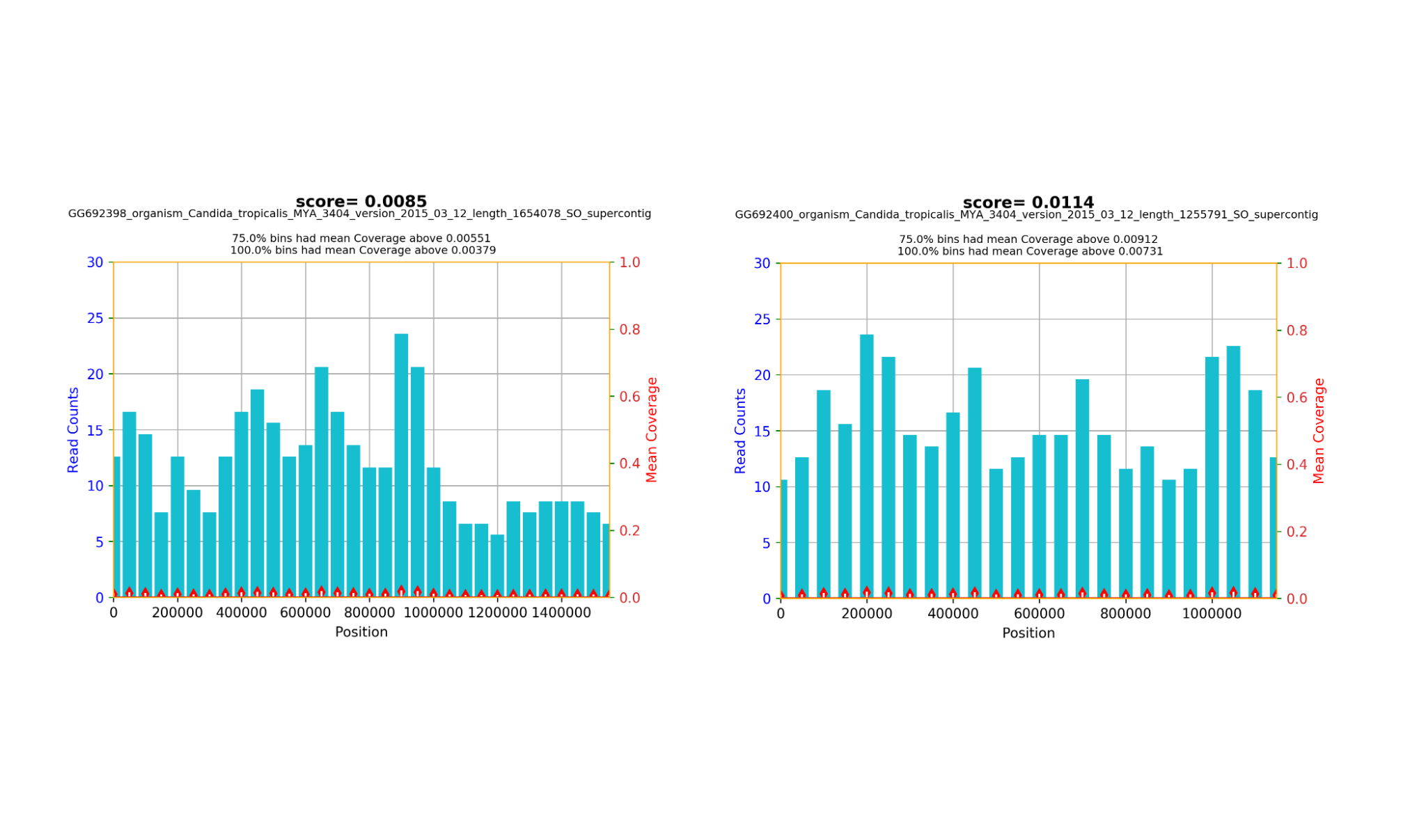
**Supplementary Figure S4** **Reads from a skin swab were mapped to a fungus from the Wochenende reference genome.** These reads were mapped with high mapping quality to all *Candida tropicalis* supercontigs, providing a rare example of a fungus in this metagenome. Fungi are generally difficult to reliably locate in metagenomes because of low abundance, poor reference genomes and wet lab sampling bias due to their highly resistant physical structures. Fungi remain rare in our experience of hundreds of particularly airway metagenomes, despite frequent reanalysis. It is not unexpected to find *C. tropicalis* at higher biomass in a skin sample, as opposed to our usual lung samples, but demonstrates our pipeline’s utility in locating eukaryotes.

# Supplementary Results

**Supplementary Table S1** **Results from Kaiju on the same mock community dataset SRR11207337 analyzed in Figure 2.** Eight bacteria should be present at 12%, with two fungi at 2% each. Results were only at genus level and are therefore reported here.

| **Taxon name** | **Percent reads assigned** |
| --- | --- |
| Salmonella | 8.52 |
| Listeria | 7.67 |
| Bacillus | 7.13 |
| Lactobacillus | 6.04 |
| Pseudomonas | 5.21 |
| Escherichia | 2.78 |
| Staphylococcus | 2.75 |
| Enterococcus | 1.07 |
| Not_assigned | 51.69% |

## Single, paired end or long read read configurations

In the clinic, cost plays a significant role in the decision-making process. While paired end reads lead to better mapping quality and therefore more precise and accurate alignments, the difference frequently did not justify the significantly increased price. Paired end reads do not indicate that more molecules have been sequenced, so the derived read counts of organisms present are not high in absolute number, but are shifted and are in some cases more highly confident. This phenomenon is taken to an extreme with long reads from the Oxford Nanopore and Pacific Biosciences platforms. While the mappings are typically extremely confident (data not shown), the absolute number of assigned reads tends to be low. This is especially relevant to the typical lung microbiomes analyzed here, where 90-95% of reads are typically derived from the human host.

We note that added value is more likely to be present in performing replicate samples from more samples, or sequencing more microbiomes, since the assemblages are typically highly variable. In clinical metagenomics, researchers and physicians are primarily concerned with identifying the high biomass species, and not the tail of potentially present rare and exotic organisms, which are unlikely to be of great clinical importance.
